# Supplementary material for: Methylomic signatures of tau and amyloid-beta in transgenic mouse models of Alzheimer’s disease neuropathology
Source: NPJ Dement. 2026 Apr 7;2(1):23. doi: 10.1038/s44400-026-00074-y (PMC13056554; doi:10.1038/s44400-026-00074-y)
Supplement: Supplementary file 1 — Supplementary Information [file 44400_2026_74_MOESM1_ESM.docx]

**Figure S1: Genomic distribution of DNA methylation sites included in the final RRBS dataset.** Summary of ChIPseeker annotations across all DNA methylation sites profiled using RRBS in rTg4510 (n = 31 WT, n = 30 TG) and J20 entorhinal cortex tissue (n = 32 WT, n = 31 TG).

**
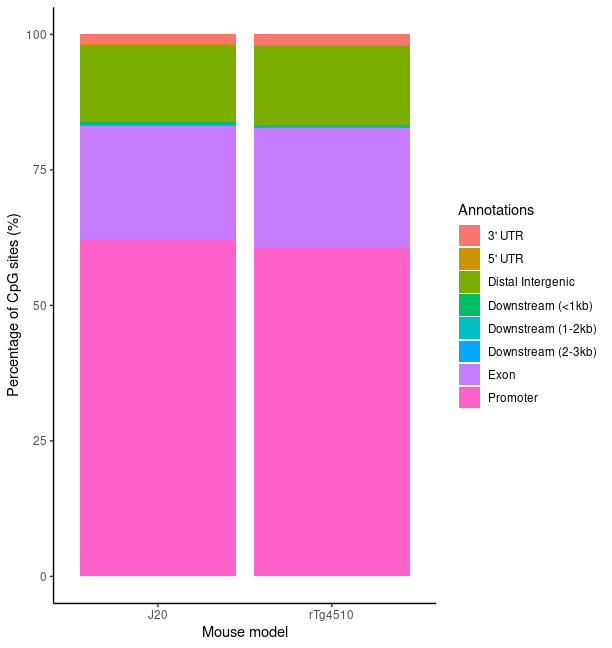
**

**Figure S2: High correlation of DNA methylation levels measured using RRBS and Illumina DNAm arrays across sites profiled using both technologies.** Shown are density plots of the mean DNA methylation values of all overlapping sites profiled using the Illumina mammalian methylation array and RRBS in the **(A)** rTg4510 entorhinal cortex (n = 61) and **(B)** J20 entorhinal cortex (n = 63).

A B


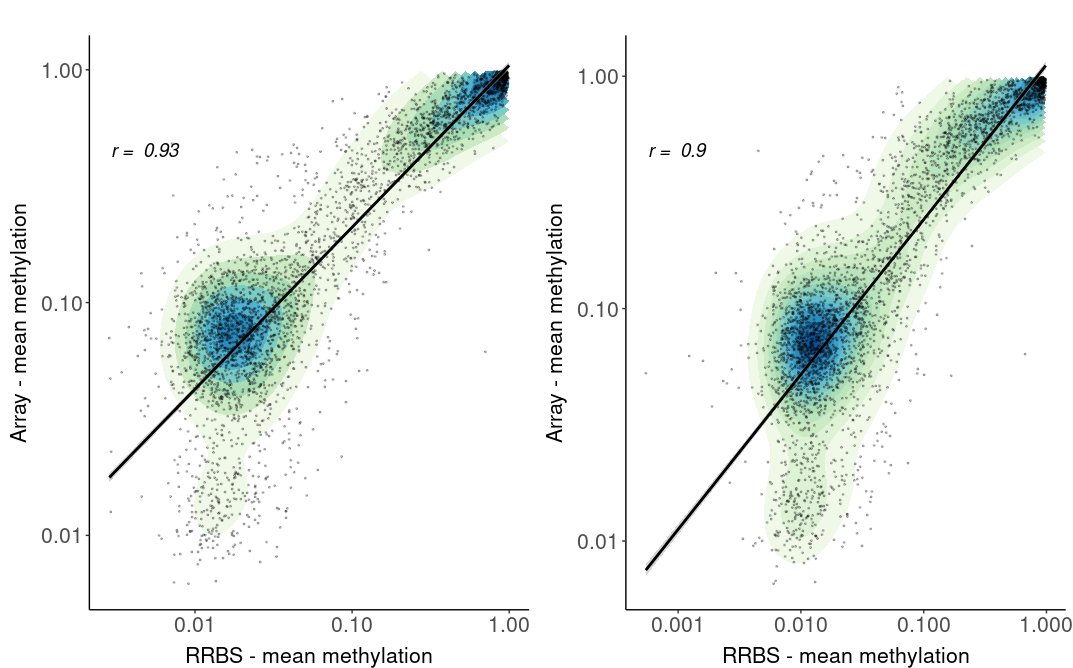


**Figure S3: Differentially methylated positions annotated to *Mapt, Prn/Prnp, Ncapg2, Fgf14* in rTg4510 TG mice.** Shown are the gene tracks and differentially methylated sites annotated to **(A)** *Mapt* (chr11:104318231, effect size = -1.04, FDR = 3.73E-30), **(B)** *Prn/Prnp* (n = 4 CpG sites, chr2:131910162 - chr2:131910201, mean effect size = 1.15), **(C)** *Ncapg2* (chr12:116425797, effect size = -0.19, FDR = 1.17E-2) and **(D)** *Fgf14* (chr14:124676565, effect size = 0.54, FDR = 1.89E-6) between WT (black) and rTg4510 TG (blue) mice.

**
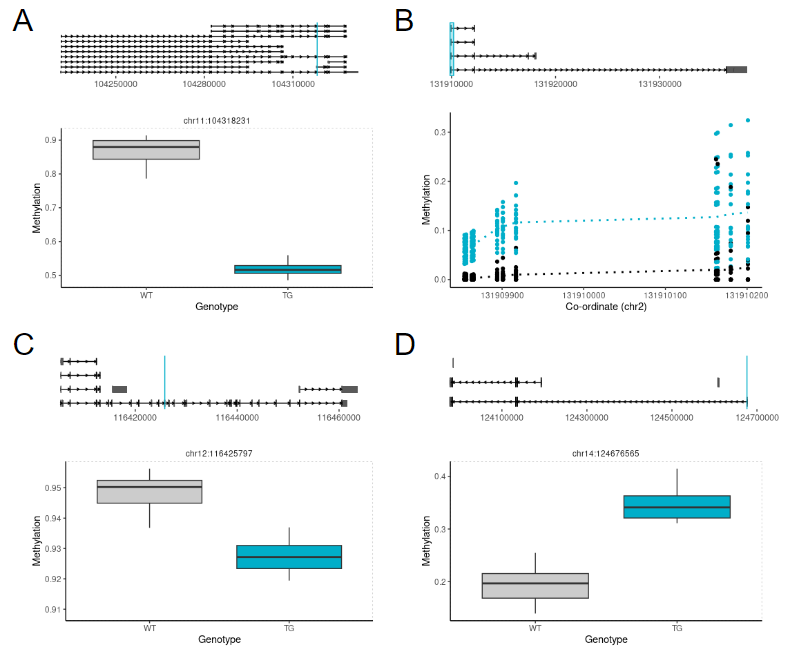
**

**Figure S4: Pyrosequencing validation of *Prn/Prnp* DMPs associated with rTg4510 genotype.** Shown are DNA methylation estimates determined from bisulfite pyrosequencing of a subset of DMPs annotated to *Prn/Prnp* in the rTg4510 entorhinal cortex (chr2:131910162: t-test P = 4.31E-25; chr2:131910164: t-test P = 6.70E-33; chr2:131910180: t-test P = 1.80E-36; chr2:131910201: t-test P = 1.59E-39) validating the results observed from RRBS (n = 4 CpG sites, chr2:131910162 - chr2:131910201, mean effect size = 1.15, Empirical Brown’s method: P = 7.00E-5).


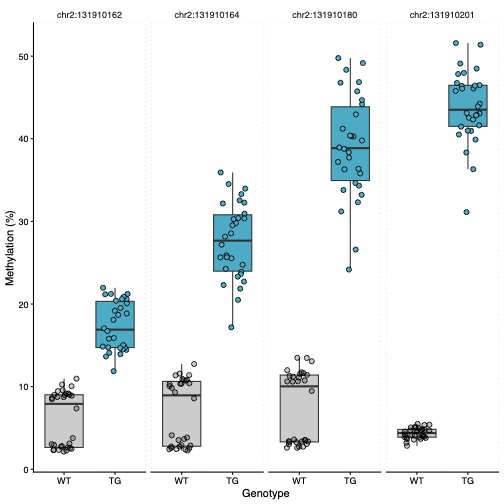


**Figure S5: Examples of differentially expressed genes annotated to rTg4510 genotype-associated DMPs in the entorhinal cortex.** Gene expression data for all genes annotated to genotype-associated DMPs is given in **Table S3**. Shown are examples of genes annotated to two of the 10 top-ranked rTg4510 DMPs highlighting differential expression in TG mice compared to WT mice for *Arsi* (P = 1.76E-02) and *Dab2ip* (P = 7.54E-03).

**
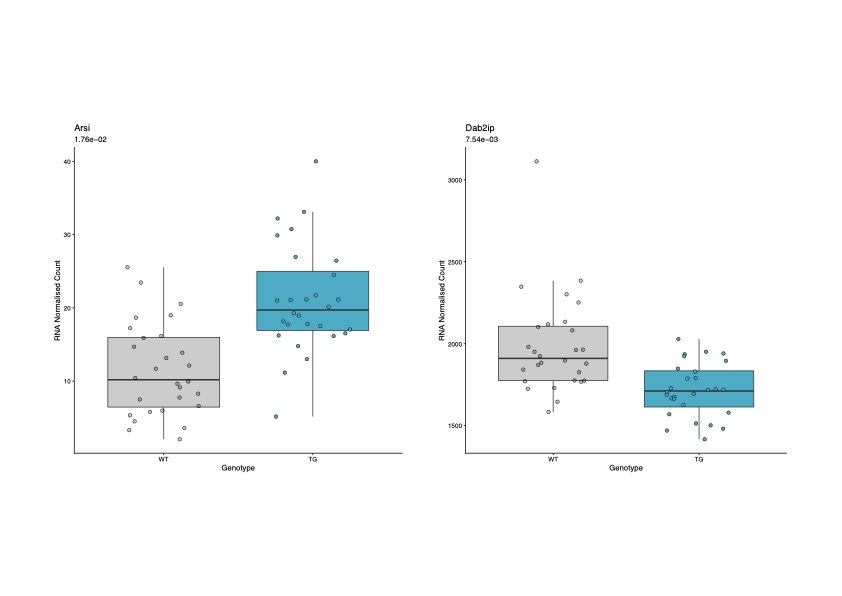
**

**Figure S6: Representative immunohistochemistry images showing accumulation of tau pathology in rTg4510 transgenic (TG) mice compared with wild-type (WT) control mice at 2, 4, 6, and 8 months of age.**

**
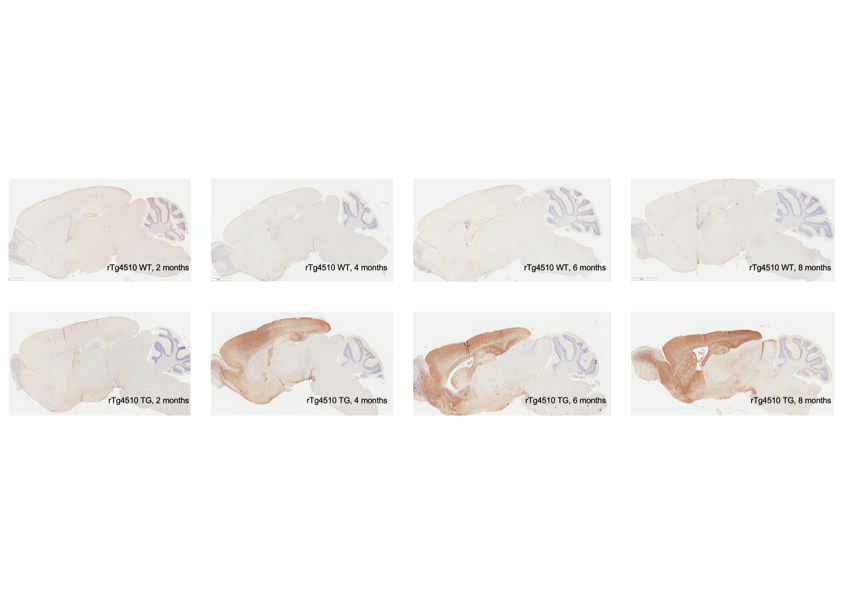
**

**Figure S7: Examples of sites at which DNA methylation shows an interaction between age and genotype in rTg4510 mice.** Shown are the top-ranked interaction effects amongst pathology-associated sites annotated to **(A)** *Kirrel3* (chr9:34618186, interaction FDR = 5.80E-08) and **(B)** *Rbm33* (chr5:28352553, interaction FDR = 5.80E-08).

**
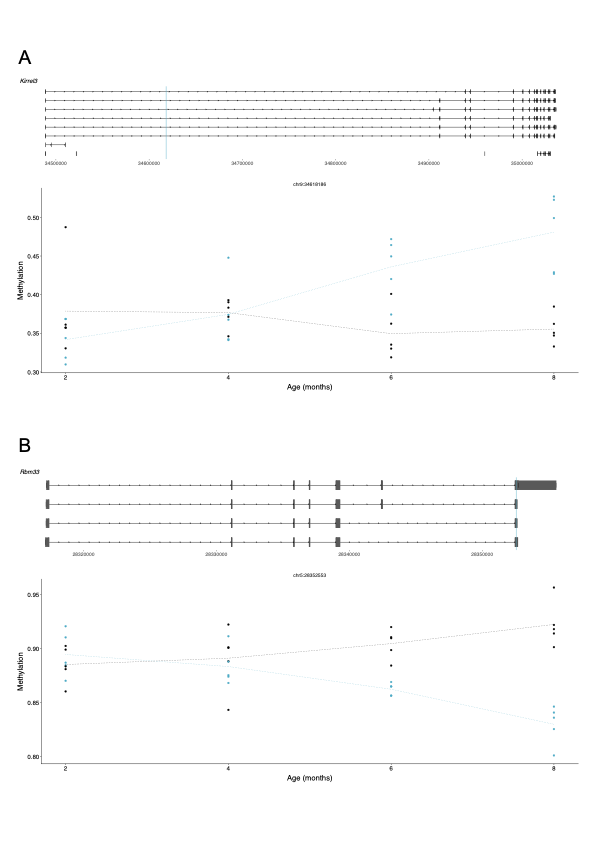
**

**Figure S8: The top-ranked differentially expressed gene annotated to a J20-associated DMP was *Slc6a11*.** Gene expression data for all genes annotated to genotype-associated DMPs is given in **Table S3**.

**
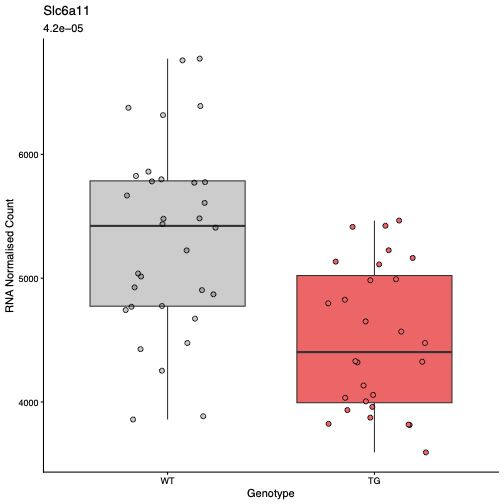
**

**Figure S9: Representative immunohistochemistry images showing progressive accumulation of amyloid pathology in J20 TG mice compared with WT mice at 6, 8, 10, and 12 months of age.**

**
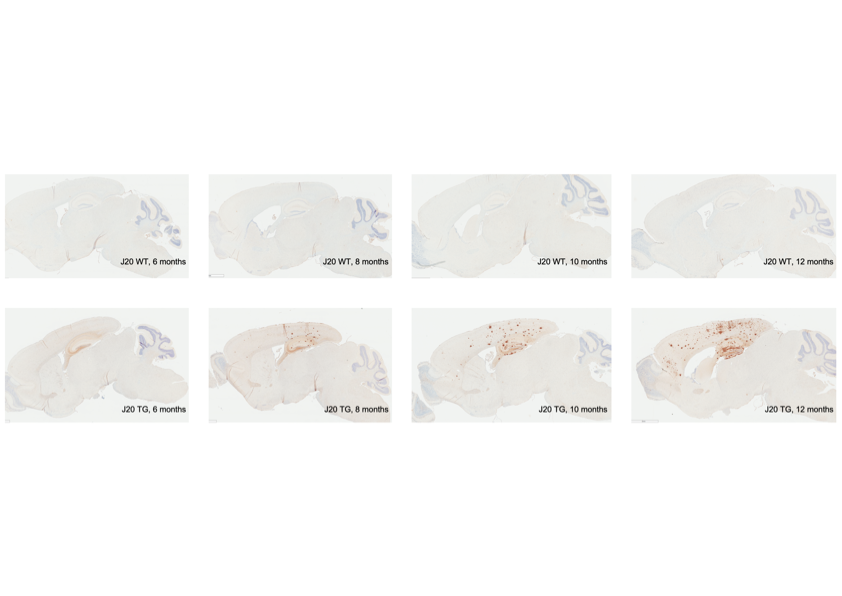
**

**Figure S10: DNA methylation changes associated with pathology, but not genotype, are strongly correlated between entorhinal cortex and hippocampus in rTg4510 mice.** Using sites profiled in both the entorhinal cortex (ECX) and hippocampus (HIP) with the Illumina mammalian DNA methylation array, we assessed the concordance of DNA methylation (DNAm) differences across brain regions. **(A)** Genotype-associated DNAm differences between rTg4510 transgenic (TG) and wild-type (WT) mice show little correlation between ECX and HIP. In contrast, **(B)** DNAm differences associated with tau pathology in rTg4510 TG mice are highly correlated across the two brain regions. Panels **(C)** and **(D)** show the corresponding relationships restricted to sites identified as significantly differentially methylated positions (DMPs) in both regions. Pearson correlation coefficients are shown for each comparison.

**
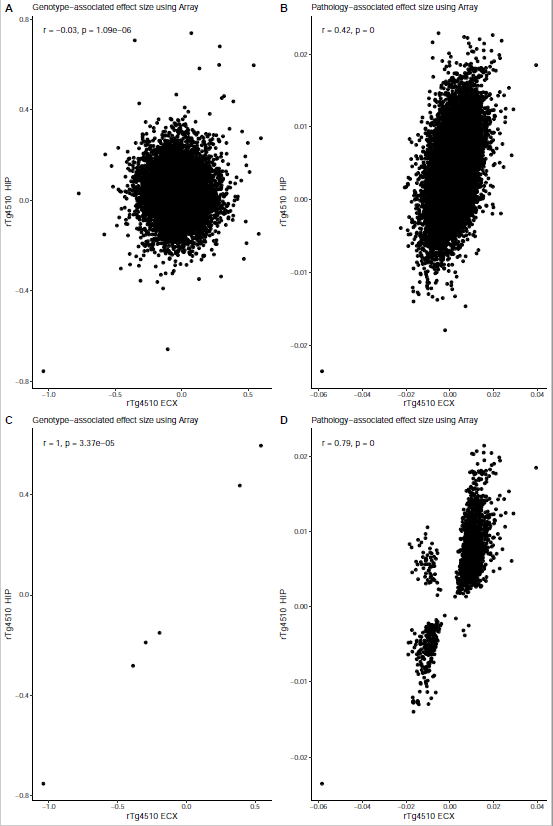
**

**Figure S11: Shared genotype-associated differentially methylated sites in the entorhinal cortex and hippocampus of rTg4510 mice.** Shown are representative differentially methylated positions (DMPs) associated with rTg4510 genotype in transgenic (TG) mice, identified in both the entorhinal cortex (ECX) and hippocampus (HIP). Panels display DMPs annotated to **(A)** *Dcaf5* (chr12:80436248), **(B)** *Satb1* (chr17:51746925), **(C)** *Cltc* (chr11:8670046), **(D)** *Mapt* (chr11:104318231), **(E)** *Ncapg2* (chr12:116425797), and **(F)** *Fgf14* (chr14:124676565), illustrating overlapping genotype-associated DNA methylation changes across the two brain regions.


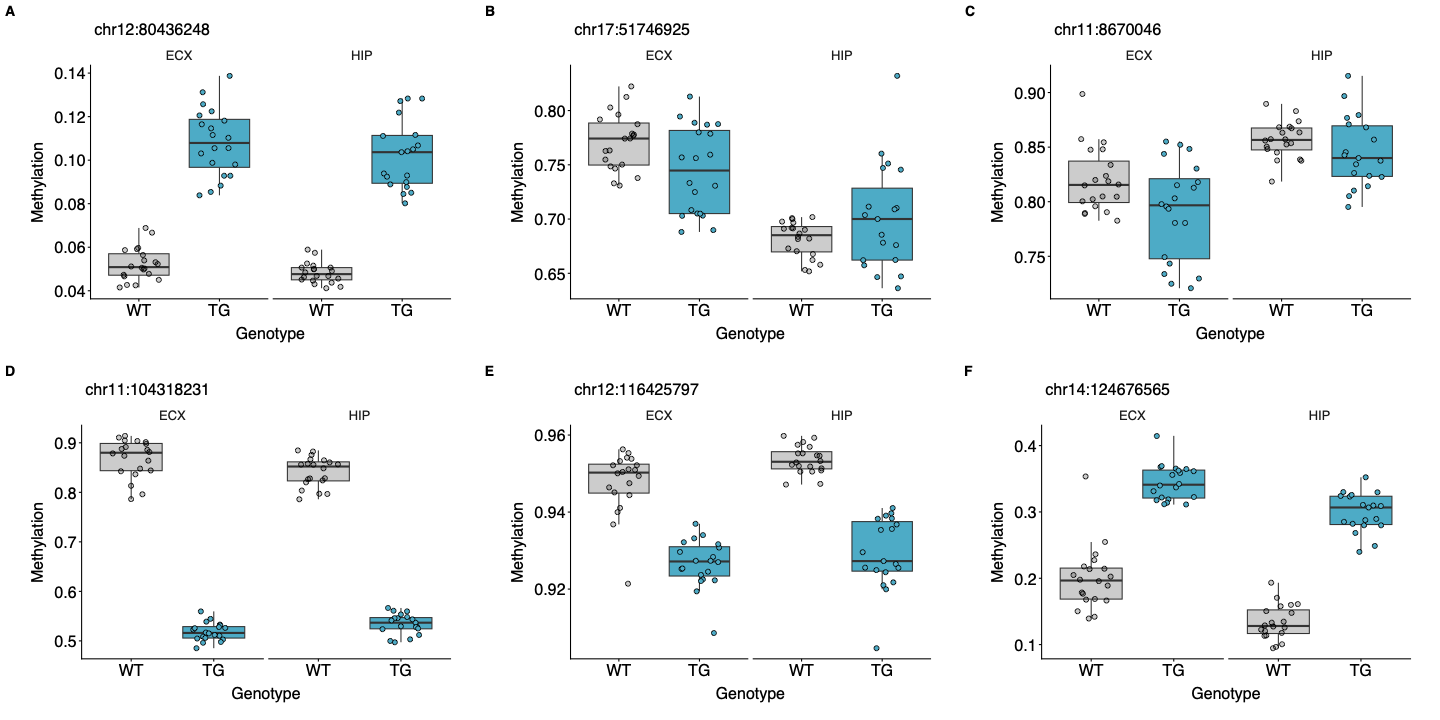


**Figure S12: Genotype- and pathology-associated DNA methylation changes are not correlated between entorhinal cortex and hippocampus in J20 mice.** Using sites profiled in both the entorhinal cortex (ECX) and hippocampus (HIP) with the Illumina mammalian DNA methylation array, we assessed the concordance of DNA methylation (DNAm) differences across brain regions in J20 mice. No correlation was observed between ECX and HIP for either **(A)** genotype-associated DNAm differences or **(B)** pathology-associated DNAm differences. Consistent with this, no CpG sites significantly associated with pathology were shared between the J20 entorhinal cortex and hippocampus.

**
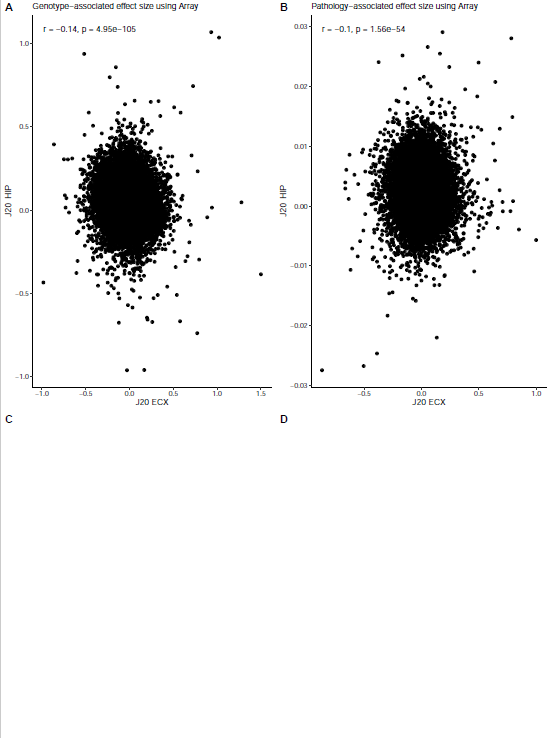
**

**Figure S13: Epigenetic age determined using epigenetic clock calibrated on mouse cortex.** Shown are scatter plots of the epigenetic age of the **(A)** rTg4510 entorhinal cortex (r = 0.89, P = 1.52E-14), **(B)** rTg4510 hippocampus (r = 0.87, P = 7.39E-14), **(C)** J20 entorhinal cortex (r = 0.81, P = 2.1E-10) and **(D)** J20 hippocampus (J20: r = 0.90, P = 4.65E-15). There is evidence for significantly accelerated epigenetic age in the hippocampus in rTg4510 TG mice.


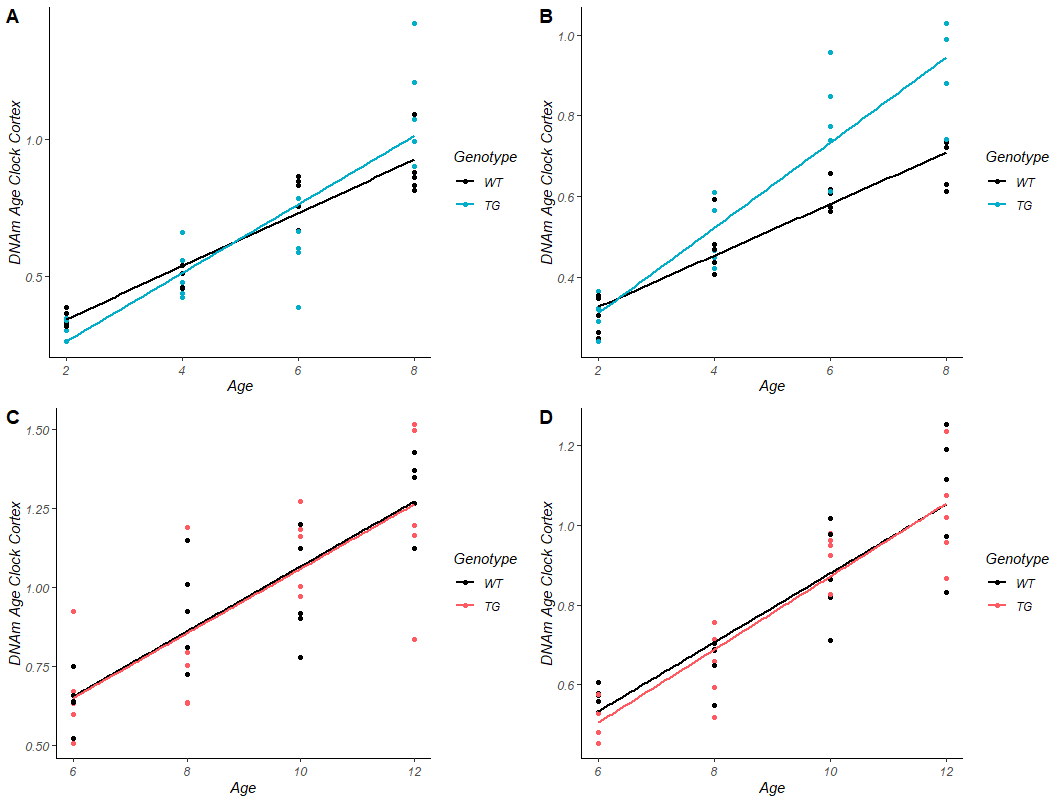


**Figure S14: DNA methylation differences annotated to *Prdm16/PRDM16* in both rTg4510 ECX and J20 ECX.** Shown are the gene tracks and differentially-methylated positions annotated to *Prdm16* **(i)** between WT and rTg4510 TG mice (chr4:154640585, effect size = 1.42, FDR = 3.49E-2; chr4:154640557, effect size = 1.41, FDR = 3.97E-2; chr4:154346846, effect size = -1.42, FDR = 4.97E-2), and **(ii)** WT and J20 TG mice (chr4:154519364, effect size = 2.12, FDR = 3.86E-2). Black, blue and red dots refer to WT, rTg4510 TG, J20 TG respectively, and lines on the tracks refer to the location of CpG sites.


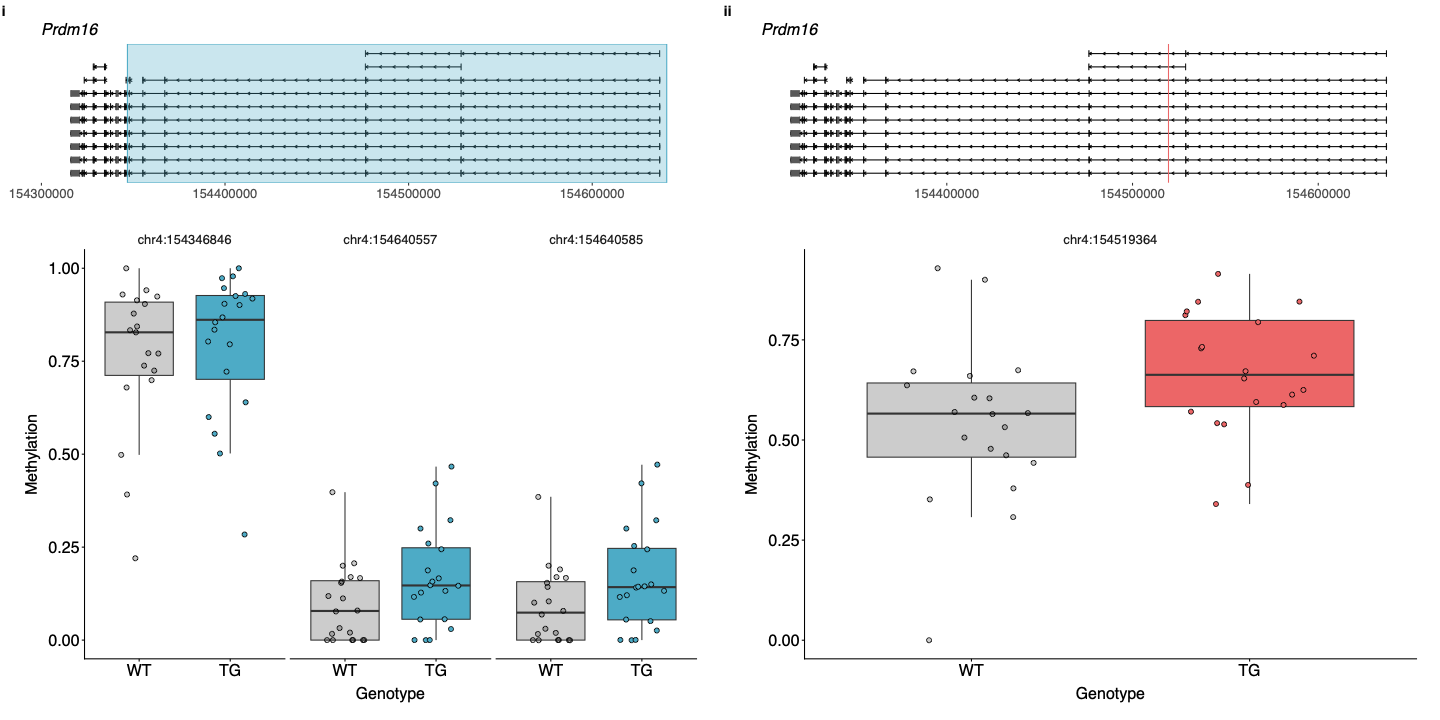


**Supplementary Data Legends**

**Data S1. Overview of samples used in this study**Neuropathology (columns F and G) refers to immunohistochemistry data from Castanho et al. 2020 (J20 amyloid, rTg4510 tau) where the burden of tau or amyloid neuropathology is expressed as a percentage affected of each brain area.

**Data S2. Site-specific entorhinal cortex DNA methylation results for rTg4510 mice**Tabulated are sites identified as significantly differentially methylated (FDR < 0.05) in analyses of either genotype, pathology or interaction (genotype x age) effects.

**Data S3. Entorhinal cortex gene expression results for genotype-associated DMPs in rTg4510 mice**

**Data S4. Functional enrichment analyses of significant genes annotated to DMPs associated with rTg4510 and J20 genotype**Tabulated are the results from functional enrichment analysis of the differentially methylated sites associated with tau and amyloid pathology in the rTg4510 and J20 entorhinal cortex respectively.

**Data S5. Site-specific entorhinal cortex DNA methylation results for J20 mice.**Tabulated are sites identified as significantly differentially methylated (FDR < 0.05) in analyses of either genotype, pathology or interaction (genotype x age) effects.

**Data S6. Entorhinal cortex gene expression results for genotype-associated DMPs in J20 mice**

**Data S7. Overlapping entorhinal cortex genotype-associated DMPs in rTg4510 and J20

Data S8. Differentially methylated sites associated with genotype and levels of tau pathology in rTg4510 hippocampus**Tabulated are the sites at which DNA methylation is significantly associated (FDR < 0.05) with either genotype or levels of tau pathology in rTg4510 hippocampus tissue. Also highlighted are sites where DNA methylation is significantly associated (FDR < 0.05) with genotype and pathology in matched entorhinal cortex (ECX) samples.

**Data S9. Differentially methylated sites associated with genotype and levels of amyloid-beta pathology in J20 hippocampus**Tabulated are the sites at which DNA methylation is significantly associated (FDR < 0.05) with either genotype or levels of Aβ pathology in J20 hippocampus tissue. Also highlighted are sites where DNA methylation is significantly associated (FDR < 0.05) with genotype and pathology in matched entorhinal cortex (ECX) samples..

**Data S10. Comparison of DNA methylation differences associated with human AD-associated pathology and tau pathology in rTg4510 cortex**This table lists genes annotated to CpG sites showing significant differential DNA methylation (FDR < 0.05) in the cortex of rTg4510 mice. Included are sites associated with (i) differences between rTg4510 transgenic (TG) and wild-type (WT) mice (genotype effect), and (ii) levels of tau pathology within rTg4510 TG mice. The HumanTauPathology column is marked TRUE for genes that were also annotated to sites associated with significant tau pathology–related differential methylation in human post-mortem cortex, as measured by Braak stage. Human tau-associated differentially methylated positions (DMPs) were obtained from a cross-cortical meta-analysis and met Bonferroni-corrected significance (P < 1.24E-07)^1^.

**Data S11. Comparison of DNA methylation differences associated with human AD-associated pathology and amyoid-beta pathology in J20**This table lists genes annotated to CpG sites showing significant differential DNA methylation (FDR < 0.05) in the cortex of J20 mice. Included are sites associated with (i) differences between J20 transgenic (TG) and wild-type (WT) mice (genotype effect), and (ii) levels of amyloid pathology within rTg4510 TG mice. The HumanAmyloidPathology column is marked TRUE for genes that were also annotated to sites associated with significant amyloid pathology–related differential methylation in human post-mortem cortex, as measured by Thal phase. Human differentially methylated positions (DMPs) were obtained from a cross-cortical meta-analysis and met Bonferroni-corrected significance (P < 1.24E-07)^1^. **References**

1. [Shireby, G. *et al.* DNA methylation signatures of Alzheimer’s disease neuropathology in the cortex are primarily driven by variation in non-neuronal cell-types. *Nat. Commun.* **13**, 1–14 (2022).](http://paperpile.com/b/OXFC6h/dUxQ)
